# Compression of room impulse responses for compact storage and fast low-latency convolution

**DOI:** 10.1186/s13636-024-00363-5

**Published:** 2024-09-13

**Authors:** Martin Jälmby, Filip Elvander, Toon van Waterschoot

**Affiliations:** 1 Department of Electrical Engineering (ESAT/STADIUS), KU Leuven, Leuven, Belgium; 2 Department of Information and Communications Engineering, Aalto University, Espoo, Finland

**Keywords:** Low-rank modeling, Room impulse responses, Convolution, Tensor decomposition

## Abstract

Room impulse responses (RIRs) are used in several applications, such as augmented reality and virtual reality. These applications require a large number of RIRs to be convolved with audio, under strict latency constraints. In this paper, we consider the compression of RIRs, in conjunction with fast time-domain convolution. We consider three different methods of RIR approximation for the purpose of RIR compression and compare them to state-of-the-art compression. The methods are evaluated using several standard objective quality measures, both channel-based and signal-based. We also propose a novel low-rank-based algorithm for fast time-domain convolution and show how the convolution can be carried out without the need to decompress the RIR. Numerical simulations are performed using RIRs of different lengths, recorded in three different rooms. It is shown that compression using low-rank approximation is a very compelling option to the state-of-the-art Opus compression, as it performs as well or better than on all but one considered measure, with the added benefit of being amenable to fast time-domain convolution.

**Supplementary information:**

The online version contains supplementary material available at 10.1186/s13636-024-00363-5.

## Introduction

Modeling the acoustics of a room as a linear time-invariant system, the room impulse response (RIR) describes the impact of the room on an acoustic excitation signal, from a certain source position to a certain receiver position. The availability of the RIR, or an accurate estimate thereof, is imperative to a multitude of acoustic signal processing tasks, such as source localization [1], speech dereverberation [2], auralization [3, 4], source separation [5], listening room compensation [6], and echo cancelation [7]. There are several ways of modeling the RIR. Among the more popular ones are the infinite impulse response (IIR) (see, e.g., [8–11]) and finite impulse response (FIR) (see, e.g., [8, 12]) models. The IIR model offers the possibility of a more compact representation, however with the downside of possible difficulties estimating the filter parameters [13], and potential issues with instability [14]. The FIR model is simple and straightforward, but with the disadvantage that comparatively many coefficients are needed to accurately represent the RIR [13]. For example, for an office-sized room, the FIR model can be several thousands of taps long [2]. A concert hall, on the other hand, can have a reverberation time of a couple of seconds [15], which at a sampling rate of 48 kHz yields an RIR with a length on the order of $10^5$ samples. This can be prohibitive from both a memory requirement and computational complexity point for view, when using the RIR for convolution [16–18].

In recent years, archaeoacoustics and the cultural heritage preservation of acoustic scenes has gained increased interest from the research community, see, e.g., [19] and references therein. However, in order to faithfully reconstruct the sound field in a room, the spatial resolution of the grid of measurements needs to be on the order of 10 cm [20]. Considering that the RIR depends on both the source and receiver position, even for a small room, the number of required source/receiver configurations for which the RIR has to be measured and stored will be in the millions, hence amounting to hundreds of gigabytes of data for the acoustic representation of a single room, indicating a significant need for compact representations of RIRs.

The computational challenges posed by long RIRs are particularly apparent in acoustic signal processing applications requiring low input-output latency, such as virtual video conferencing [21], augmented/mixed reality [22] and virtual reality [3, 23], sound zone control [24, 25], network music performance [26], and artificial reverberation [27]. In this paper, we consider rendering techniques based on convolution, highlighting the need for fast, low-latency convolution with long RIRs.

Ever since the works of Cooley and Tukey [28], and Stockham [29], a popular approach has been to carry out convolution in the frequency domain. With the help of the convolution theorem, which states that (circular) convolution in the (discrete) time domain is equivalent to point-wise multiplication in the (discrete) frequency domain, one is able to significantly reduce the computational burden of convolution in most cases, owing to the computational efficiency of the fast Fourier transform (FFT) algorithm. Frequency-domain convolution has since been further improved by methods such as overlap-add (OLA) and overlap-save (OLS), and partitioned convolution. For an overview of these methods, see, e.g., [30, 31]. A drawback of frequency-domain convolution is, however, that it is block-based, and therefore inevitably introduces latency. Further, partitioned frequency-domain filters are subject to restrictions with regard to assembling them into networks of filters (in parallel or serial structure), which is not the case for time-domain filters [32]. Another possible way to attempt to speed up the computations is by perceptual convolution [33]. There, the convolution is simplified, based on a perceptual criterion. The number of frequency-domain multiplications and the memory storage are reduced by up to 60%, without considerable quality degradation. Another path is optimization with respect to processor architecture and the use of graphics processing units (see, e.g., [34] and references therein). Yet another approach is to effectively shorten the RIR by treating the different parts of the RIR separately. For example, in [35], convolution is carried out for the first parts of the RIR, corresponding to the direct component and early reflections. The late reverberation, however, is modeled as a velvet noise sequence, yielding a very sparse FIR filter. Instead of being convolved with the sparse FIR, the input signal is propagated in the delay line of the filter, and only the samples coinciding with a non-zero component of the sparse FIR are added together to yield the output. Similarly, hybrid reverberation can also be achieved by considering convolutional reverb for the early parts of the RIR and a feedback delay network synthesizing of the reverberation tail [36].

In this paper, we consider RIR compression and fast low-latency time-domain convolution based on three different methods; truncation, (hard) thresholding, and low-rank approximation. The exploitation of the (approximate) low-rank structure of reshaped RIRs is something we have considered in previous work. The physical motivation for it, and its applicability to real-life RIRs, was demonstrated in [37]. How the low-rank structure can be exploited when estimating RIRs from noisy input-output relations was shown in [38] and the simultaneous compression of multiple RIRs was considered in [39]. Atkins et al. showed in [40] how this low-rank structure can be exploited in time-domain convolution, an idea we extended upon in recent work [41]. Jaderberg et al. showed in [42] how speeding up convolutional neural networks can be done by leveraging low rank, but the authors consider dimensions no higher than 3.

The contribution of this paper is threefold. Firstly, we provide an extensive comparison of the aforementioned compression methods, with respect to several objective quality measures, both channel-based and signal-based. Secondly, we propose an approximate fast time-domain convolution method based on *N*-D low-rank tensor approximation of an RIR. This yields lower computational complexity than traditional time-domain convolution and lower latency than FFT-based fast convolution. Thirdly, we show how the problem of compression and fast time-domain convolution can be handled within the same framework. This comes with the major advantage that the compressed RIR does not need to be decompressed before it can be used for convolution.

This paper is organized as follows: first, Sect. 1 is concluded with an introduction of the notation used throughout the paper, as well as the introduction of the signal model. In Sect. 2, the different RIR approximations considered for RIR compression are introduced. In Sect. 3, convolution by low-rank approximation is introduced. Sect. 4 introduces the different objective quality measures that will be used for evaluation. Numerical results are presented in Section 5, and finally, conclusions are presented in Sect. 6.

### Notation and signal model

We denote scalars, vectors, matrices, and tensors by lowercase (e.g., *h*), bold lowercase (e.g., $\textbf{h}$), bold uppercase (e.g., $\textbf{H}$), and calligraphic letters (e.g., $\mathcal {H}$), respectively. Sets are also denoted by calligraphic letters, but it will be clear from context what is considered. The selection of one or several elements from a vector, matrix, or tensor will be denoted by square brackets, e.g., ${\textbf{H}[m:n,j]}$ is a vector containing the *m*th till the *n*th element of the *j*th column of $\textbf{H}$, and the hat symbol, $\hat{\cdot }$, indicates an approximated quantity. The symbol $\circ$ denotes the outer product, i.e., $(\textbf{x}_1 \circ \textbf{x}_2 \circ \dots \circ \textbf{x}_D)[j_1,j_2, \dots , j_D] = \textbf{x}_1[j_1] \textbf{x}_2[j_2] \dots \textbf{x}_D[j_D]$, ( : ) denotes vectorization of a matrix or a tensor, and $\lfloor \cdot \rfloor$ denotes the flooring operation.

We consider a discrete-time RIR *h*(*k*), for $k = 0, 1, ..., n_{h} -1$, arranged in a vector $\textbf{h} \in \mathbb {R}^{n_{h}}$, as well as a discrete-time signal *x*(*k*), for ${k = 1,2, \dots , n_{x}}$, arranged in the vector $\textbf{x} \in \mathbb {R}^{n_{x}}$. The convolution of these vectors yields the discrete-time output

1 $$\begin{aligned} y(k) = \sum \limits _{n = 0}^{n_{h} - 1} h(n) x(k-n), \end{aligned}$$

for ${k = 1,2, \dots , n_{y}}$, with corresponding vector $\textbf{y} \in \mathbb {R}^{n_{y}}$, where $n_{y} = n_{h} + n_{x} -1$. Generally, throughout this paper, an element is considered to be 0, if the index is out of its defined range, equivalent to appropriate zero-padding.

## Room impulse response compression

We will consider three different RIR approximations for RIR compression and compare them to a state-of-the-art compression benchmark.

### Compression by truncation

Firstly, we consider an RIR compressed by *truncation*, $\hat{\textbf{h}}_{\text {T}}$, where

2 $$\begin{aligned} \hat{\textbf{h}}_{\text {T}}(n) = \left\{ \begin{array}{l} \textbf{h}(n), n \le n_{\text {T}} \\ 0, n > n_{\text {T}} \end{array}\right. \end{aligned}$$

for some $n_{\text {T}} \in \mathbb {N}, n_{\text {T}} \le n_{h}$. This method is amenable to accelerated convolution, as the length of the impulse response is shortened, decreasing the number of multiply-add instructions per output sample from $n_{h}$ to $n_{\text {T}}$.

### Compression by thresholding

Secondly, we consider an RIR compressed by *thresholding*^1^, $\hat{\textbf{h}}_{\text {K}}$, defined as

3 $$\begin{aligned} \hat{\textbf{h}}_{\text {K}}(n) = \left\{ \begin{array}{l} \textbf{h}(n), n \in \mathcal {K}_{n_{\text {k}}} \\ 0, n \notin \mathcal {K}_{n_{\text {k}}} \end{array}\right. \end{aligned}$$

where $\mathcal {K}_{n_{\text {k}}}$ is the set of indices of the $n_{\text {k}}$, in absolute value, largest elements of $\textbf{h}$. Also this RIR approximation method yields a possibly faster convolution. As many of the elements of $\hat{\textbf{h}}_{\text {K}}$ are zero, these do not have to be considered in the convolution. For a sparse impulse response $\hat{\textbf{h}}_{\text {K}}$, we can define the convolution between $\hat{\textbf{h}}_{\text {K}} \in \mathbb {R}^{n_{h}}$ and $\textbf{x} \in \mathbb {R}^{n_{x}}$ as

4 $$\begin{aligned} y(k) = \sum \limits _{n \in \mathcal {K}_{n_{\text {k}}}} \hat{\textbf{h}}_{\text {K}}(n) x(k-n). \end{aligned}$$

This reduces the number of multiply-add instructions per output sample from $n_{h}$ to $n_{\text {k}}$. The argument could be made that the positions of the non-zero components need to be stored, and that is something that needs to be taken into account as well. However, whereas the coefficients themselves are floating numbers, the positions are integers, taking up significantly less space. Therefore, the impact of having to store the positions was ignored when considering the compression of thresholding.

### Compression by low-rank approximation

Lastly, we consider an RIR compressed by *low-rank* approximation, $\hat{\textbf{h}}_{\text {LR}}$. Assuming $n_{h} = n_{s_1} n_{s_2}$, with $n_{s_1}, n_{s_2} \in \mathbb {N}$, the RIR $\textbf{h} \in \mathbb {R}^{n_{h}}$ can be reshaped into a matrix $\textbf{H} \in \mathbb {R}^{n_{s_1} \times n_{s_2}}$,

5 $$\begin{aligned} \textbf{H} = \left[ \begin{array}{cccc} h(1) & h(n_{s_1} + 1) & \dots & h(n_{s_1}(n_{s_2}-1)+1) \\ \vdots & \vdots & & \vdots \\ h(n_{s_1}) & h(2n_{s_1}) & \dots & h(n_{h}) \end{array}\right] . \end{aligned}$$

With the use of the singular value decomposition (SVD) ${\textbf{H} = \textbf{U} \varvec{\Sigma } \textbf{V}^T}$ and assuming the singular values in $\varvec{\Sigma }$ are arranged in non-increasing order, we can then make a rank-*R* approximation of $\textbf{H}$,

6 $$\begin{aligned} \hat{\textbf{H}}_{2\text {D}} = \textbf{U}[:,1:R] \varvec{\Sigma }[1:R,1:R] \textbf{V} [:,1:R]^T. \end{aligned}$$

Finally, $\hat{\textbf{h}}_{2\text {D}} = \hat{\textbf{H}}_{2\text {D}}(:)$. Similarly, assuming $n_{h} = \prod _{d=1}^{D} n_{s_d}$, $n_{s_d} \in \mathbb {N}$, the vector $\textbf{h}$ can be reshaped into a tensor ${\mathcal {H} \in \mathbb {R}^{n_{s_1} \times n_{s_2} \times \dots \times n_{s_D}}}$, of arbitrary dimension *D*, where $n_{s_d}$ denotes the size of the *d*th dimension and the rank of a tensor is defined as the smallest number of rank-1 tensors needed to generate the tensor $\mathcal {H}$ as their sum. In a similar fashion as to the matrix, we can then make a rank-*R* approximation $\hat{\textbf{h}}_{\text {LR}}$ of $\mathcal {H}$. For this we will be using a (canonic) polyadic decomposition (see, e.g., [43] and references therein). This is done using the high-level function *cpd* of the Matlab toolbox Tensorlab [44]. Subsequently, $\hat{\textbf{h}}_{\text {LR}} = \hat{\textbf{h}}_{\text {LR}}(:)$. We will, in addition to aforementioned $\hat{\textbf{h}}_{2\text {D}}$, consider low-rank approximation of 3-D and 5-D tensors, denoted $\hat{\textbf{h}}_{3\text {D}} = \hat{\textbf{H}}_{3\text {D}}(:)$ and $\hat{\textbf{h}}_{5\text {D}} = \hat{\textbf{H}}_{5\text {D}}(:)$, respectively. The absence of a 4-D tensor approximation is explained in Sect. 5. Also the low-rank approximation method allows for fast time-domain convolution, which we have explored in recent work for up to three dimensions [41]. Here we will extend this idea to tensors of arbitrary dimensions. This will be further explained in Sect. 3.

### Compression benchmark: Opus

**Fig. 1:**
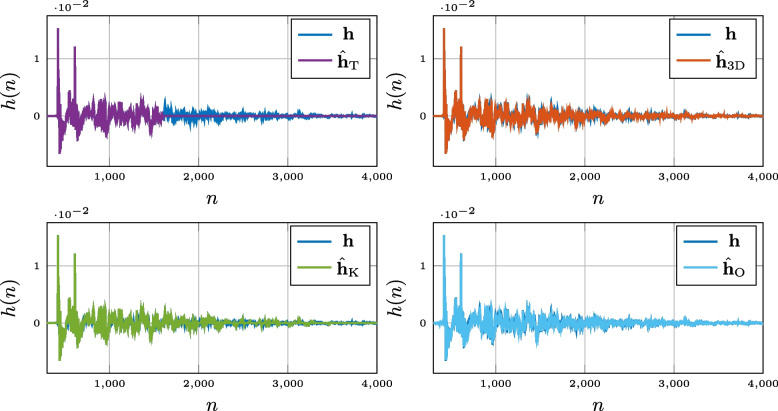
Illustrative simulation example of four RIR compression methods considered in this paper: truncation ($\hat{\textbf{h}}_{\text {T}}$), thresholding ($\hat{\textbf{h}}_{\text {K}}$), low-rank approximation ($\hat{\textbf{h}}_{\text {3D}}$), and Opus ($\hat{\textbf{h}}_{\text {O}}$). Whereas early reflections in the RIR are well preserved by each of the compression methods, the RIR tail is entirely or partially removed by the truncation and thresholding methods, respectively

The three methods proposed above, truncation, thresholding, and low-rank approximation, will be compared to the state-of-the-art Opus interactive speech and audio codec [45, 46]. The Opus codec is created from two core technologies: Skype’s SILK codec [47], based on Linear Prediction Coding (LPC), and Xiph.Org’s CELT codec [48, 49], based on the Modified Discrete Cosine Transform (MDCT). The idea behind this construction is that LP is considered to code low frequencies more efficiently, whereas for music and higher speech frequencies, MDCT is superior. The double layers allow Opus to achieve higher quality for a wide range of audio. The Opus codec was created for, and has previously mainly been considered for, speech and music, but it has recently also gained attention as a possible way to compress RIRs [50]. A possible explanation for why the Opus codec performs relatively well for RIR compression can be found in the distinct spectral characteristics of room acoustics in the lower vs. higher frequency ranges, which align well with the double-layer structure of Opus. In the lower frequency range, the spectral behavior is often dominated by room modes [51], which can be accurately represented by means of autoregressive models [52, 53], the model parameters of which are estimated with LPC. In the higher frequency range, the room modes become more densely spaced and exhibit a less narrowband response due to high-frequency wall absorption. This results in a high-frequency room magnitude response that is not so much characterized by individual magnitude peaks but rather by a smooth spectral envelope, which is exactly the type of spectral behavior for which the CELT codec has been conceived [48]. In this work, the Opus encoding was done using Matlab’s *audiowrite*. It should be noted that although Opus shrinks the file size of the stored RIR, the number of coefficients remains the same. The RIR compressed by Opus, that will be denoted $\hat{\textbf{h}}_{\text {O}}$, is therefore, to the best of the authors’ knowledge, not amenable to fast time-domain convolution. In order to give the reader a feel for the different approximations, an example RIR, taken from [54], and a selection of the compressed RIRs obtained with the different compression methods, at a compression rate (see (32)) of 0.8, are displayed in Fig. 1.

## Convolution by low-rank approximation

Accelerating convolution by exploiting low-rank approximations was first considered by Atkins et al. in [40]. The authors there considered a low-rank approximation of a matricization of the RIR, using the SVD. In recent work, [41], we have extended this idea to a three-dimensional tensorization of the RIR. We will here show how this idea can be further extended to a tensorization of arbitrary dimension. We will first demonstrate the 2-D case presented in [38], and then explain the extension to a tensor of arbitrary dimension.

### Partitioned truncated SVD filter

Assuming $n_{h} = n_{s_1} n_{s_2}$, for $n_{s_1}, n_{s_2} \in \mathbb {N}$, an output sample *y*(*k*) of the convolution in (1) can be written as

7 $$\begin{aligned} y(k) = \sum \limits _{j = 1}^{n_{s_2}} \textbf{x}_k^{(j)^T} \textbf{h}^{(j)}, \end{aligned}$$

where

8 $$\begin{aligned} \textbf{h}^{(j)} \triangleq \left[ \begin{array}{ccc} h( (j-1) n_{s_1} )&\ \dots&\ h( j n_{s_1} - 1) \end{array}\right] \in \mathbb {R}^{n_{s_1}} \end{aligned}$$

and

9 $$\begin{aligned} \textbf{x}^{(j)}_k \triangleq \left[ \begin{array}{ccc} x( k - (j - 1)n_{s_1})&\ \dots&\ x(k - j n_{s_1} + 1) \end{array}\right] \in \mathbb {R}^{n_{s_1}}, \end{aligned}$$

for $j = 1, 2, \dots , n_{s_2}$. Instead of as in (1) writing *y*(*k*) as the inner product of two vectors of length $n_{h} = n_{s_1} n_{s_2}$, it is in (7) written as the sum of $n_{s_2}$ inner products of vectors of length $n_{s_1}$. Further, the RIR $\textbf{h}$ can be reshaped into a matrix $${\textbf{H} = \begin{bmatrix} \textbf{h}^{(1)}&\dots&\textbf{h}^{(n_{s_2})} \end{bmatrix} \in \mathbb {R}^{n_{s_1} \times n_{s_2}}}$$. For now we are going to assume that this matrix is rank-1, i.e., it can be written as the outer product $\textbf{H} = \textbf{s}_1 \circ \textbf{s}_2$, with $\textbf{s}_1 \in \mathbb {R}^{n_{s_1}}$ and $\textbf{s}_2 \in \mathbb {R}^{n_{s_2}}$. Under this assumption, we have that

10 $$\begin{aligned} \textbf{H} = \left[ \begin{array}{cccc} \textbf{s}_1 \textbf{s}_2[1]&\ \textbf{s}_1 \textbf{s}_2[2]&\ \dots&\ \textbf{s}_1 \textbf{s}_2[n_{s_2}] \end{array}\right] , \end{aligned}$$

i.e., the *j*th column of $\textbf{H}$, corresponding to $\textbf{h}^{(j)}$, is the vector $\textbf{s}_1$ scaled by $\textbf{s}_2[j]$, $j = 1,2, \dots , n_{s_2}$. While any rank-decomposition could be used, we consider the case when $\textbf{s}_1$ and $\textbf{s}_2$ are the left and right singular vectors, respectively, corresponding to the largest singular value of $\textbf{H}$. Further, the following property is readily verified,

11 $$\begin{aligned} \textbf{x}_k^{(j)} = \textbf{x}_{k + a n_{s_1}}^{(j + a)}, a \in \mathbb {Z}. \end{aligned}$$

Because of (10) and (11), only the first inner product of the sum in (7) has to be computed per output sample *k*, the other inner products of the sum, i.e., $\textbf{x}_k^{(j)^T} \textbf{s}_1 = \textbf{x}_{k - n_{s_1}}^{(j - 1)^T} \textbf{s}_1$, for $j = 2, \dots , n_{s_2}$, have already been computed for a previous time sample, and can therefore be fetched from memory and multiplied with the appropriate entry from $\textbf{s}_2$,

12 $$\begin{aligned} y(k) =\left( \textbf{x}_k^{(1)^T} \textbf{s}_1 \right) \textbf{s}_2[1] + \sum \limits _{j = 2}^{n_{s_2}} \underbrace{\left( \textbf{x}_k^{(j)^T} \textbf{s}_1 \right) }_{\text {Fetch from memory}} \textbf{s}_2[j]. \end{aligned}$$

This reduces the number of multiplications per sample to be carried out, from ${n_{h} = n_{s_1} n_{s_2}}$ to ${n_{s_1} + n_{s_2}}$. These ideas can be extended to a matrix $\textbf{H}$ of arbitrary rank *R*. Instead of $\textbf{H}$ being just the outer product of two vectors, it is now a sum of *R* outer products,

13 $$\begin{aligned} \textbf{H} = \textbf{S}_1 \textbf{S}_2^T = \sum \limits _{r = 1}^R \textbf{S}_1[:,r] \circ \textbf{S}_2[:,r] = \sum \limits _{r = 1}^R \textbf{S}_1[:,r] \textbf{S}_2[:,r]^T, \end{aligned}$$

for $\textbf{S}_1 \in \mathbb {R}^{n_{s_1} \times R}$, and $\textbf{S}_2 \in \mathbb {R}^{n_{s_2} \times R}$. Equation (12) can now be extended to

14 $$\begin{aligned} y(k) = & \sum \limits _{r = 1}^R \left( \left( \textbf{x}_k^{(1)^T} \textbf{S}_1[:,r] \right) \textbf{S}_2[1,r] +\right. \nonumber \\ & \sum \limits _{j = 2}^{n_{s_2}} \left.\underbrace{\left( \textbf{x}_k^{(j)^T} \textbf{S}_1[:,r] \right) }_{\text {Fetch from memory}} \textbf{S}_2[j,r] \right) \end{aligned}$$

where only *R* inner products have to be computed for each time sample. Similar to (12), this reduces the number of multiplications to ${R(n_{s_1} + n_{s_2})}$. Much like $\textbf{s}_1$ and $\textbf{s}_2$ were the left and right singular vectors, respectively, corresponding to the largest singular values, we consider the case where the columns of $\textbf{S}_1$ and $\textbf{S}_2$ are the right and left singular vectors, respectively, corresponding to the *R* largest singular values. For more details, the reader is referred to [40].

### Fast time-domain convolution by tensor approximation

We are now ready to extend these ideas to a tensor of arbitrary dimension. Assuming $\textbf{h} \in \mathbb {R}^{n_{h}}$, with $n_{h} = \prod _{d = 1}^{D} n_{s_d}$, for $n_{s_1}, n_{s_2}, \dots , n_{s_D} \in \mathbb {N}$, let $\textbf{h}$ be reshaped into a tensor $\mathcal {H} \in \mathbb {R}^{n_{s_1} \times n_{s_2} \times \dots \times n_{s_D}}$, and assume that $\mathcal {H}$ is of rank *R*. Then, analogously to (13),

15 $$\begin{aligned} \mathcal {H} = \sum \limits _{r=1}^R \textbf{S}_1[:,r] \circ \textbf{S}_2[:,r] \circ \dots \circ \textbf{S}_D[:,r], \end{aligned}$$

where $\textbf{S}_d \in \mathbb {R}^{n_{s_d} \times R}$, $d = 1,2, \dots , D$, and in analog to (10), but with arbitrary dimension and rank, we have that

16 $$\begin{aligned} \mathcal {H}[:, j_2, j_3, \dots , j_D] = \sum \limits _{r=1}^R \textbf{S}_1[:,r] \textbf{S}_2[j_2,r] \dots \textbf{S}_D[j_D,r]. \end{aligned}$$

The equality of (11) can be generalized according to

17 $$\begin{aligned} \textbf{x}_k^{(j_2, j_3, \dots , j_{D})} = \textbf{x}_{k + \sum _{d=2}^{D} a_d \prod _{p=1}^{d-1} n_{s_p}}^{(j_2 + a_2, j_3 + a_3, \dots , j_{D} + a_{D})}, \end{aligned}$$

where $\textbf{x}_k^{(j_2, j_3, \dots , j_{D})} \in \mathbb {R}^{n_{s_1}}$ is a vector containing the $n_{s_1}$ latest samples of $\textbf{x}$, in reversed order, starting at ${x(k - \sum _{d = 2}^D (j_d - 1) \prod _{p = 1}^{d - 1} n_{s_p})}$, and $a_2, a_3, \dots , a_D \in \mathbb {Z}$. While verifying (17) can seem like a daunting task, it becomes clearer when considering the indices of the first entry of the vectors on the left and right hand side of (17), respectively,

18 $$\begin{aligned} & k - \sum \limits _{d = 2}^D (j_d - 1) \prod _{p = 1}^{d - 1} n_{s_p} = \nonumber \\ & k + \sum \limits _{d=2}^{D} a_d \prod _{p=1}^{d-1} n_{s_p} - \sum \limits _{d = 2}^D (j_d +a_d - 1) \prod _{p = 1}^{d - 1} n_{s_p}. \end{aligned}$$

The pattern from (7) extends to

19 $$\begin{aligned} y(k) = \sum \limits _{j_2 = 1}^{n_{s_2}} \dots \sum \limits _{j_D = 1}^{n_{s_D}} \textbf{x}_k^{(j_2, j_3, \dots , j_D)^T} \textbf{h}^{(j_2, j_3, \dots , j_D)}, \end{aligned}$$

where ${\textbf{h}^{(j_2, j_3,\dots , j_D)} = \mathcal {H}[:, j_2, j_3, \dots , j_D]}$ is a vector containing $n_{s_1}$ consecutive elements of $\textbf{h}$, starting at ${h(\sum _{d=2}^D (j_d - 1) \prod _{p = 1}^{d - 1} n_{s_p})}$. Subsequently, the property of (14) is generalized to

20 $$\begin{aligned} & y(k) = \sum \limits _{r=1}^R \left( \textbf{x}_k^{(1, \dots , 1)^T} \textbf{S}_1 [:,r] \textbf{S}_2[1,r] \dots \textbf{S}_D[1,r] +\right. \nonumber \\ & \left. \sum \limits _{j_2 = 2}^{n_{s_2}} \dots \sum \limits _{j_D = 2}^{n_{s_D}} \underbrace{\left( \textbf{x}_k^{(j_2, \dots , j_D)^T} \textbf{S}_1 [:,r] \right) }_{\text {Fetch from memory}} \textbf{S}_2[j_2,r] \hspace{-0.6 mm} \dots \textbf{S}_D[j_D,r] \right) \end{aligned}$$

with a corresponding structure of what has to be computed and what can be fetched from memory. Similarly to the previous case, we have a reduction in complexity. Only *R* inner products of length $n_{s_1}$ have to be computed for each time index *k*, reducing the number of multiplications to ${R \sum _{d = 1}^{D} n_{s_d}}$. When naively implemented, the sum in (20) will yield many superfluous operations, where one of the vectors contains only zeros. To fully exploit the structure of the RIR, and to maximize efficiency, it is therefore important to keep track of which operations actually need to be carried out and keep the number of multiplications with zeros to a minimum. We here propose an explicit algorithm.

Let ${\mathcal {H} = \sum _{r=1}^R \textbf{S}_1[:,r] \circ \textbf{S}_2[:,r] \circ \dots \circ \textbf{S}_D[:,r]}$, where ${\mathcal {H} \in \mathbb {R}^{n_{s_1} \times n_{s_2} \times \dots \times n_{s_D}}}$, and $\textbf{S}_d \in \mathbb {R}^{n_{s_d} \times R}$, for $d = 1,2, \dots , D$. The operator $\mathcal {I}: \mathbb {R}^n \rightarrow \mathbb {R}^n$ denotes the reversion of the order of the elements in a vector, i.e., $$\mathcal {I}(\textbf{x}) = \left[ \begin{array}{cccc} x(n_{x}) \ &x(n_{x} - 1)&\ \dots \ &x(1) \end{array}\right] ^T$$, and $\textbf{0}_n \in \mathbb {R}^n$ is a vector of zeros. The foundation of the algorithm is that, for each *k*, we compute the *R* necessary inner products, store the resulting values to memory and add these to *y*(*k*) with appropriate scaling by the corresponding elements of $\mathcal {H}$. Next, the remaining non-zero inner products in the sum of (20) are fetched from memory, scaled by the corresponding entry of $\mathcal {H}$ and added to *y*(*k*). The fast low-latency convolution algorithm by low-rank tensor approximation is summarized in Algorithm 1. A few remarks regarding Algorithm 1, for providing intuition as well as clarity, are in order:

- New inner products need to be computed and stored to memory as long as $k \le n_{s_1} + n_{x} - 1$, this is done within the if-statement starting at line 5.
- Within the for-statement starting at line 14 the old inner products are fetched from memory and added to the output.
- On line 15, for $d = 2$, the upper limit of $\prod _{p = 2}^{d-1} n_{s_p}$ is lower than the lower limit, in which case, by convention, $\prod _{p = 2}^{1} n_{s_p} = 1$.

**Figure:**
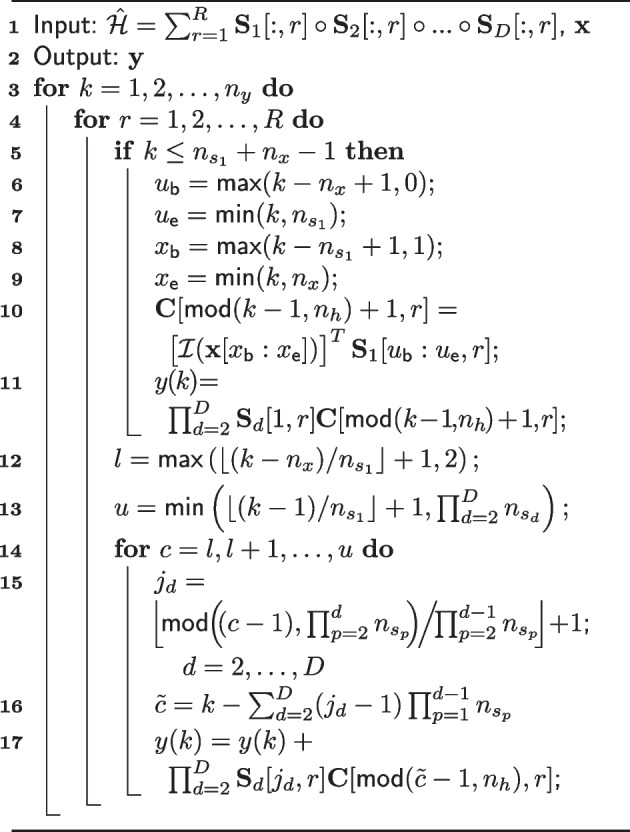
**Algorithm 1** Fast low-latency convolution by low-rank tensor approximation

### Complexity

**Fig. 2:**
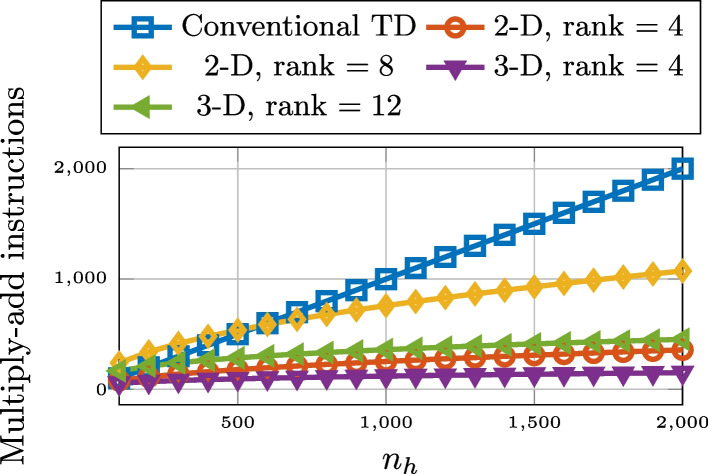
Computational complexity of proposed convolution

By the authors of [40], it was noted that an output sample *y*(*k*) requires $R(n_{s_1} + n_{s_2})$ multiply-add instructions, in the two-dimensional case, compared to the $n_{h} = n_{s_1} n_{s_2}$ multiply-add instructions of conventional FIR filter convolution. The computational complexity for a general, *D*-dimensional tensorization is a generalization of the one in [40], and amounts to ${R \sum _{d= 1}^{D} n_{s_d}}$ multiply-add instructions, as compared to $n_{h} = \prod _{d = 1}^{D} n_{s_d}$ multiply-add instructions of conventional FIR filter convolution. Further, as the contribution to the end result of the entries in the sum of (20) are independent from each other, it is possible to perform these computations in parallel. To provide some intuition, an example is shown in Fig. 2. Here the complexity of traditional time-domain convolution is, for varying values of $n_{h}$, compared to that of the proposed algorithm for the case of square 2-D matricization and 3-D tensorizations of rank 4 and 12.

The two-dimensional algorithm from [40] requires a memory of size $R (n_{s_1} + n_{s_2} + n_{h}) + n_{s_1}$ variables, compared to $2 n_{h}$ for a conventional FIR filter. For the proposed method, it is ${R(\sum _{d=1}^{D} n_{s_d} + n_{h}) + n_{s_1}}$, i.e., also the memory requirement for the proposed method is a generalization of the one in [40].

## Objective quality measures

Audio technology can generally be designed to be either physically motivated or perceptually motivated. Physically motivated techniques are typically computationally intensive, in the attempt to physically accurately represent the sound field. Perceptually motivated systems are in general less computationally demanding, as they aim only to be accurate enough for human perception [18]. The physical accuracy of low-rank approximations of RIRs was evaluated in [37]; in this work, we aim to investigate the perceptual accuracy of compression by low-rank approximation and the other aforementioned compression methods. In this section, we describe a variety of parameters regarding the perception of room acoustics and corresponding objective measures. These measures can be divided into two categories, channel-based objective measures and signal-based objective measures [2]. The channel-based measures concern only how well the approximation of the channel, i.e., the compressed RIR, relates to the measured channel, i.e., the RIR. Signal-based measures, on the other hand, pertain to how the approximated channel distorts the signal output, after the compressed RIR has been convolved with e.g., music or speech.

The objective of the different measures considered here differs slightly. For some of them a high value is desirable, for others a lower value is better. For most of them, however, invariance is what is sought after, i.e., that the value of the measured quantity for a compressed RIR is as close as possible to the measured quantity for the original RIR. For an easy overview for the reader, the measures considered in this paper, their definitions, whether they are channel- or signal-based, and their objectives, are recapped in Table 1.

**Table 1:** Measures

Measure	Definition	Channel/Signal	Objective
Normalized misalignment	(21)	Channel	Low
Energy decay curve	(22)	Channel	Invariance
Reverberation time $T_{60}$	(23)	Channel	Invariance
Echo density	(24)	Channel	Invariance
Early decay time (EDT)	(26)	Channel	Invariance
Center time	(27)	Channel	Invariance
TOA of direct component	(28)	Channel	Invariance
Frequency-weighted log-spectral signal distortion (SD)	(31)	Signal	Low
ViSQOLAudio	[55–57]	Signal	High

### Channel-based objective quality measures

The perhaps most obvious way to measure the quality of a compressed RIR is by the *normalized misalignment*, defined as

21 $$\begin{aligned} \mathcal {M}_{\text {dB}} \left( \hat{\textbf{h}} \right) = 20 \, \text {log}_{10} \left( \frac{\Vert \hat{\textbf{h}} - \textbf{h} \Vert _2}{\Vert \textbf{h} \Vert _2} \right) . \end{aligned}$$

The problem with this measure is, however, that it is not necessarily a good indicator of whether the compressed RIR will yield an auditory perception faithful to the original RIR.

*Reverberation time* is a well-known objective measure for room acoustics. This is the time it takes for the sound level to drop 60 dB, after a stationary sound source has been switched off, and is denoted $T_{60}$. In practice, this measure is typically estimated as double the time it takes for the sound level to drop from $-5$ to $-35$ dB [15]. Finding the time it takes for the sound level to drop a certain amount is done via the *energy decay curve* which, since the work by Schroeder [58], is most commonly found using backwards integration. As we consider discrete-time signals in this paper, the energy decay curve *D*(*n*) is found using backwards summation,

22 $$\begin{aligned} D(n) = \sum \limits _{k = n}^{n_{h}} h^2(k) = \sum \limits _{k = 0}^{n_{h}} h^2(k) - \sum \limits _{k = 0}^{n} h^2(k). \end{aligned}$$

Letting $n_{-x \text {dB}}$ denote the time sample when the energy decay curve *D*(*n*) has decreased to *x* dB below its starting value, $T_{60}$ is found as

23 $$\begin{aligned} T_{60} = 2 \left( n_{-35 \text {dB}} - n_{-5 \text {dB}} \right) f_s, \end{aligned}$$

where $f_s$ denotes sampling frequency. Reverberation can cause degraded speech intelligibility, but it is also what gives music fullness, by blending the sounds of different instruments and voices [15]. It further provides, together with the energy ratio between direct and reverberant sound and the time of arrival of the early reflections, information about the size of a space and the distance to the boundaries [59].

The *echo density* profile of an RIR is the fraction of impulse response coefficients which lie outside the standard deviation of the coefficient amplitudes, for a particular time window. A simple and robust measure for echo density was introduced by Abel et al. in [60],

24 $$\begin{aligned} \eta (n) = \frac{1/\text {erfc}\left( 1/\sqrt{2}\right) }{2 \delta + 1} \sum \limits _{k = n - \delta }^{n + \delta } w(k)\textbf{1}_{\left\{ |h(k)| > \sigma \right\} }, \end{aligned}$$

where $\text {erfc}\left( 1/\sqrt{2}\right) = 0.3173$, $2 \delta + 1$ is the window length in samples, $\textbf{1}_{\left\{ \cdot \right\} }$ is an indicator function, *w*(*k*) is a window function, for which $\sum _{k} w(k) = 1$, and

25 $$\begin{aligned} \sigma = \left[ \sum \limits _{k = n - \delta }^{n + \delta } w(k) h^2(k) \right] ^{1/2}. \end{aligned}$$

Throughout this paper, we will use a Hanning window with $\delta = 550$, when $f_s = 44.1$ kHz and $\delta = 600$ when $f_s = 48$ kHz, corresponding to a window length of 25 ms, as per the discussion in [60]. Further, we will only consider the part of the echo density profile where the entire window fits.

In reverberant music or speech, later parts of the reverberation tend to be masked by the direct and early components of the next note or syllable. Therefore, the alternative measure *early decay time* (EDT), has proved to be better correlated with reverberance, a perceptual attribute of reverberation, than reverberation time, in the aforementioned scenarios [15]. The EDT is defined as

26 $$\begin{aligned} \text {EDT} = 6 (n_{-10 \text {dB}}) f_s. \end{aligned}$$

The parameter *center time*, denoted $t_s$, describes the balance between early and late energy in the RIR [15], defined as

27 $$\begin{aligned} t_s = \frac{\sum _{k = 0}^{n_{h}}k h^2(k)}{\sum _{k = 0}^{n_{h}} h^2(k)}, \end{aligned}$$

i.e., the center of gravity of the RIR. Two other measures that are commonly mentioned in this context are *mode density* [61, 62] and *reflections density* [18, 51]. These are, however, better suited to characterize synthetically generated RIRs. As we here consider only real-life RIRs, these measures will not be considered in this paper.

The time of arrival (TOA) of the direct component, defined as

28 $$\begin{aligned} \text {TOA} = \left( \underset{n}{\mathrm {arg\,max}} \left| h(n) \right| \right) / f_s, \end{aligned}$$

is crucial in tasks such as room geometry estimation [63] and acoustic source localization [64]. How the TOA of the direct component is preserved by a compression method is not well captured by the normalized misalignment and will therefore be considered as a separate measure in Sect. 5.

For all the channel-based measures introduced above, except normalized misalignment, we aim for a minimal deviation between the compressed and original RIR measure. We will therefore, in Sect. 5, present the root-mean-square error (RMSE) for these quantities,

29 $$\begin{aligned} \text {RMSE}_g \left( \hat{\textbf{h}} \right) = \sqrt{\sum \limits _{j = 1}^{n_{\text {RIR}}} \left|g \left( \textbf{h}_j \right) - g(\hat{\textbf{h}}_j ) \right|^2 / n_{\text {RIR}}}, \end{aligned}$$

where *g* is the considered measure, and $n_{\text {RIR}}$ denotes the number of RIRs used in the evaluation. We alert the reader that we in Sect. 5 will consider RMSE in linear scale for certain measures and in logarithmic scale for other measures, depending on what best highlights the difference in performance between the considered compression methods. All considered quantities except the energy decay curve and echo density are scalar, making the computation of the RMSE straightforward. These, however, are discrete-time sequences. There the RMSE will be computed as

30 $$\begin{aligned} \text {RMSE}_{g}\left( \hat{\textbf{h}} \right) = \sqrt{\sum \limits _{j = 1}^{n_{\text {RIR}}} \Vert \textbf{h}_{g}^j - \hat{\textbf{h}}_{g}^j \Vert ^2_2 / n_{\text {RIR}} n_{g}}, \end{aligned}$$

where $$\textbf{h}_{g}^j = \begin{bmatrix} g(1),&g(2),&\dots ,&g(n_g) \end{bmatrix}^T$$ denotes the considered quantity of the *j*th RIR, and $n_g$ its length.

### Signal-based objective measures

Next, we present measures of output signal degradation. The ultimate goal of any acoustic signal enhancement or reproduction task is to achieve good signal quality. One way to measure this is by using subjective listening test. These tests are, however, expensive, tedious, and time consuming [50, 65]. Therefore, several objective measures have been developed to predict the outcome of subjective listening tests. The frequency-weighted log-spectral signal distortion (SD) [66] is a perceptually weighted objective measure of distortion of a sound signal, w.r.t. a reference signal

31 $$\begin{aligned} \text {SD}(t) = \sqrt{\int _{f_l}^{f_u} w_{\text {ERB}}(f) \left( 10 \log _{10}\frac{P_{\hat{\textbf{y}}}(f,t)}{P_{\textbf{y}}(f,t)} \right) ^2 df}, \end{aligned}$$

where $P_{\hat{\textbf{y}}}$ and $P_{\textbf{y}}$ are the short-term power spectra of $\hat{\textbf{y}} = \textbf{x} * \hat{\textbf{h}}$ and $\textbf{y} = \textbf{x} * \textbf{h}$, for a sound signal $\textbf{x}$, respectively, and $w_{\text {ERB}}$ is a frequency-weighting function, that gives equal weight to each auditory critical band between $f_l = 300$ Hz and $f_u = 6500$ Hz. In Sect. 5, we will present the mean SD for the respective scenarios.

Hines et al. introduced the Virtual Speech Quality Objective Listener (ViSQOL) [55, 56], an objective measure for predicting the subjective assessment of perceived speech quality, based on the Neurogram Similarity Index Measure (NSIM) [67]. ViSQOL was subsequently extended to ViSQOLAudio [57], to comprise not only speech, but also audio and music signals, and has shown high correlation with the subjective listening test MUSHRA [68]. Narbutt et al. have extended ViSQOL and ViSQOLAudio to AMBIQUAL [69, 70], that aims to predict not only listening quality, but also localization accuracy, for spatial audio. We do not consider spatial audio in this work and will therefore not use AMBIQUAL. In addition to the aforementioned acoustic qualities and measures, there are several other measures concerning perceived speech quality, such as PESQ [71] and POLQA [72]. These are intended to predict the perceived quality of speech, rather than audio or music, and will not be considered here.

## Numerical results

To compare the performance of the here investigated methods, we apply them to three different datasets of RIRs, with varying reverberation time. First we apply it to the single- and multichannel audio recordings database (SMARD) [54], which contains RIRs from a listening room with a reverberation time of approximately 0.15 s, sampled at 48 kHz. Next, we apply the methods to the two different datasets from the MYRiAD database [73]. The first one is from the Alamire Interactive Laboratory (AIL), which has a reverberation time of 0.5 s, and the second one is from the SONORA Audio Laboratory (SAL), with a reverberation time of 2.1 s. These are sampled at 44.1 kHz.

For the low-rank methods, the matricization or tensorization of the RIRs brings about the question of the size of the dimensions. For a *D*-dimensional tensorization, it is required that $\prod _{d = 1}^{D} n_{s_d} = n_{h}$, but this can be achieved in several different ways. The impact of the size of the dimensions is beyond the scope of this paper, and we will here present only square matricizations and tensorizations, i.e., ${n_{s_1} = n_{s_2} = \dots = n_{s_D}}$. As a consequence of this, we must have that ${n_{s_d} = \root D \of {n_{h}} \in \mathbb {N}}$. For this reason, the length of the RIRs for the different compression methods will vary slightly. We will here present the results for low-rank approximations of different dimensions, thresholding, truncation, and, as a benchmark, Opus. In order to be able to have RIR lengths in as close proximity as possible, we present low-rank approximations for $D = 2, 3, \text {and } 5$, neglecting $D = 4$, as the length of the RIR for that dimension of tensorization would differ too much from the others. The RIR lengths used for the 2-D, 3-D, and 5-D approximations are denoted $n_{h_2}$, $n_{h_3}$, and $n_{h_5}$, respectively. The RIR length used for thresholding, truncation, and Opus is denoted $n_{h}$ and will be equal to the largest of $n_{h_2}$, $n_{h_3}$, and $n_{h_5}$, for the respective scenarios. The different RIR lengths used in the simulations are found in Table 2. We alert the reader that these lengths apply to both the approximation and their respective reference RIR, as some of the objective measures introduced in Sect. 4 require that the approximated RIR and the reference RIR are of equal length. For the generation of the output signals, the compressed RIRs are convolved with 6 different 15 s snippets of anechoic music from [74]. When convolving these snippets of music with the RIRs from SMARD, the music was upsampled to 48 kHz using Matlab’s *resample*, in order to have matching sampling frequencies.

**Table 2:** RIR lengths used for the different data sets

Name	$n_{\text {RIR}}$	$n_{h_2}$	$n_{h_3}$	$n_{h_5}$
SMARD	100	$88^2 = 7744$	$20^3 = 8000$	$6^5 = 7776$
AIL	40	$181^2 = 32761$	$32^3 = 32768$	$8^5 = 32768$
SAL	20	$316^2 = 99856$	$47^3 = 103823$	$10^5 = 100000$

We denote by $\Upsilon (\hat{\textbf{h}})$ the number of coefficients needed to be stored for a certain compressed RIR $\hat{\textbf{h}}$, and remind the reader that for the low-rank approximations, $\Upsilon (\hat{\textbf{h}}) = R \sum _{d = 1}^{D} n_{s_d}$. For all the compression methods except Opus, the number of coefficients stored coincides with the number of multiply-add instructions needed to carry out time-domain convolution with the approximated RIR. For the original RIR, this number is $n_{h}$. Therefore, by

32 $$\begin{aligned} C (\hat{\textbf{h}}) = 1 - \frac{\Upsilon (\hat{\textbf{h}})}{n_{h}}, \end{aligned}$$

where $C(\hat{\textbf{h}}) \in [0, 1)$, we denote both *compression rate* and *complexity reduction*. For $C (\hat{\textbf{h}}) = 0$ there is no compression or complexity reduction, whereas for $C (\hat{\textbf{h}})$ closer to 1, the degree of complexity reduction is larger. We provide simulations in the range from $C(\hat{\textbf{h}}) = 0.7$ to $C(\hat{\textbf{h}}) = 0.95$, as these are the minimum and maximum values of compression supported by Opus, for all the sets of RIRs considered here, when using Matlab’s built-in function *audiowrite*.

RIRs should ideally be estimated from noiseless measurements, but this condition is often not met in practice [75–77]. As the RIRs used in this paper are taken from databases of real-life recorded RIRs, they will contain some measurement noise. However, to simulate a realistic environment, white Gaussian noise was added to each recorded and truncated RIR before compression and convolution. The power of the noise was adjusted to yield a signal-to-noise ratio (SNR) of 20 dB, as in [78], where

33 $$\begin{aligned} \text {SNR}_{\text {dB}} = 10 \, \text {log}_{10} \left( \frac{P_R}{P_N} \right) , \end{aligned}$$

where $P_R$ and $P_N$ denote the power of the RIR without the noise, and the power of the noise, respectively. The ground-truth values of the quantities considered in this section is computed with respect to truncated RIR, before the noise is added.

On a couple of occasions, the performance of one, or several, compression methods was significantly worse than the other methods. In those cases, these approximations have been left out of the figures, as including them would significantly impact the scaling of the figure, and prevent the reader from noticing the differences between the more competitive methods. When this has been done, remarks have been made in the corresponding subsection to alert the reader.

### Normalized misalignment

**Fig. 3:**
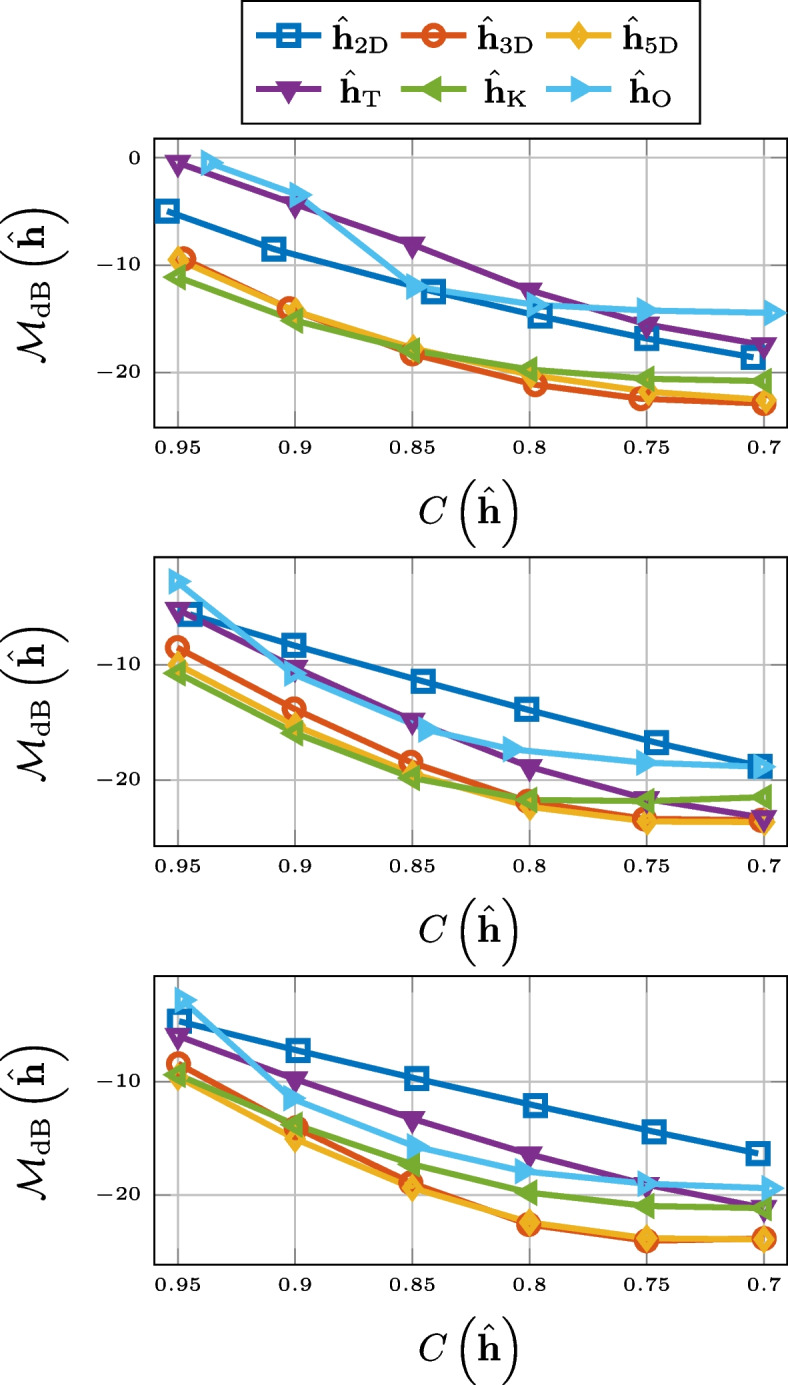
Normalized misalignment for SMARD (top), AIL (middle), and SAL (bottom) for different compression methods

As can be seen in Fig. 3, in terms of normalized misalignment for the RIR compression, truncation and 2-D matricization falls short. However, 3-D tensorization, 5-D tensorization, and thresholding are all outperforming Opus.

### Reverberation time $T_{60}$

**Fig. 4:**
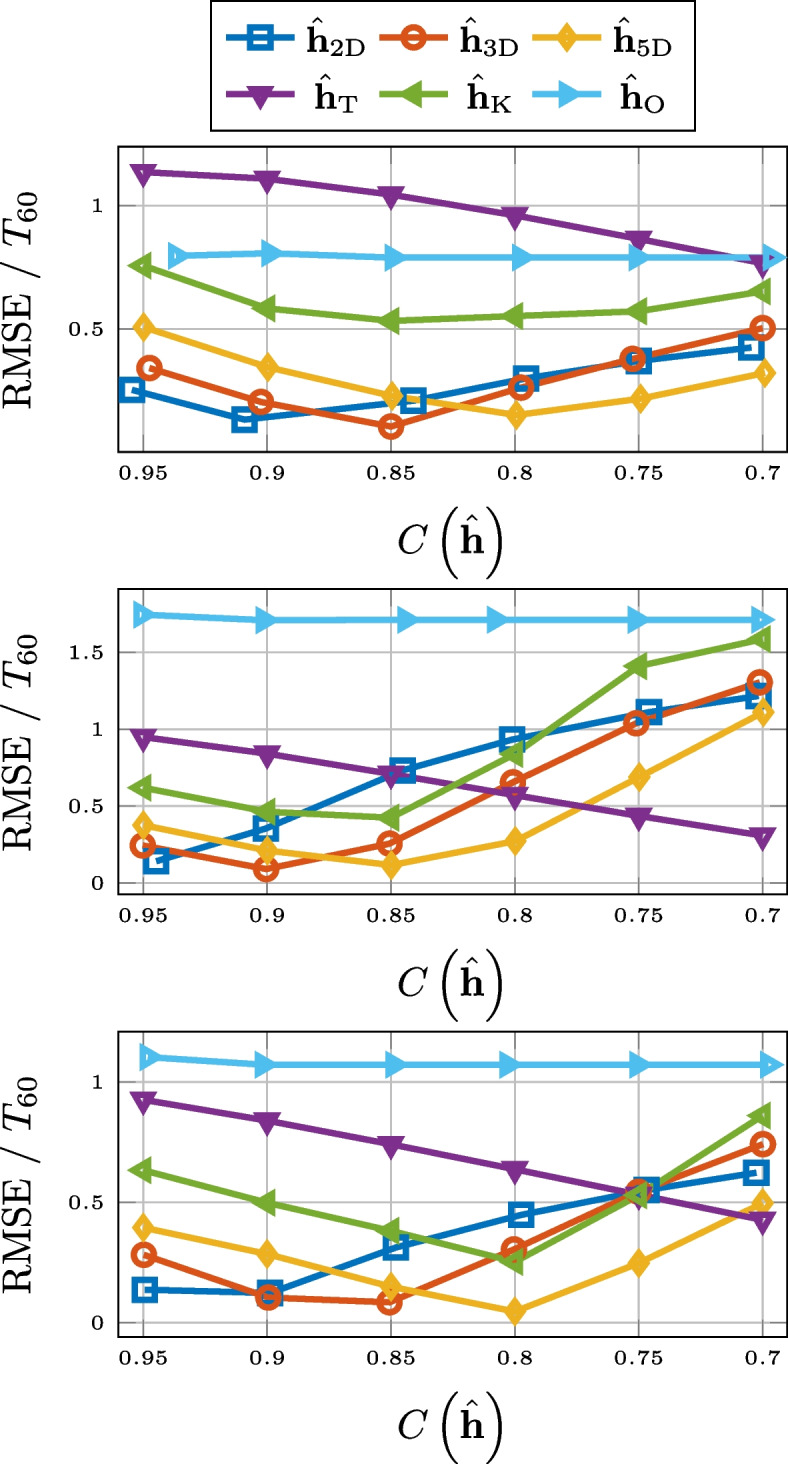
RMSE for $T_{60}$ for SMARD (top), AIL (middle), and SAL (bottom) for different compression methods

**Fig. 5:**
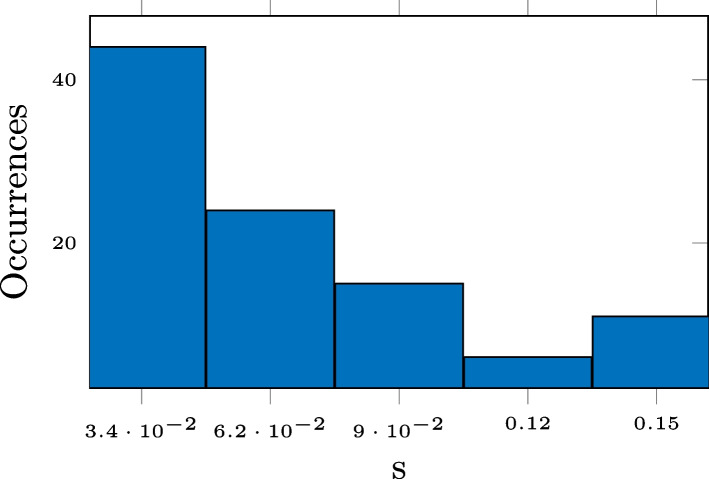
Histogram of differences in $T_{60}$ estimate, $T_{60}(\hat{\textbf{h}}_{3\text {D}}) - T_{60}(\textbf{h})$, for 3-D tensor approximation and recorded RIR

Compression based on low-rank approximation or thresholding also performs very well when it comes to the preservation of the reverberation time $T_{60}$. This is displayed in Fig. 4, where we observe a consistent outperformance of Opus. The unexpected performance deterioration for the low-rank approximation and thresholding is due to the added noise. Overestimation of $T_{60}$ for noisy RIRs is a well-known phenomenon [79, 80]. This is due to a slower drop-off of the decay curve (22). The approximations serve as denoising but for lower values of compression there is still a systematic overestimation of the reverberation time. This is illustrated in Fig. 5, where histogram of the differences between the $T_{60}$ estimates for the 3-D tensor approximation and that of the measured RIR, for the RIRs of SMARD, at the compression rate of 0.7, is displayed. We alert the reader that these are differences and not absolute differences, i.e., the fact that all numbers are positive shows the consistent overestimation. Preliminary simulations showed that this systematic overestimation could partly be alleviated by estimating the $T_{60}$ a shorter time interval, i.e., corresponding to the decay from $-5$ to $-25$ dB, but not entirely.

### Energy decay curve

**Fig. 6:**
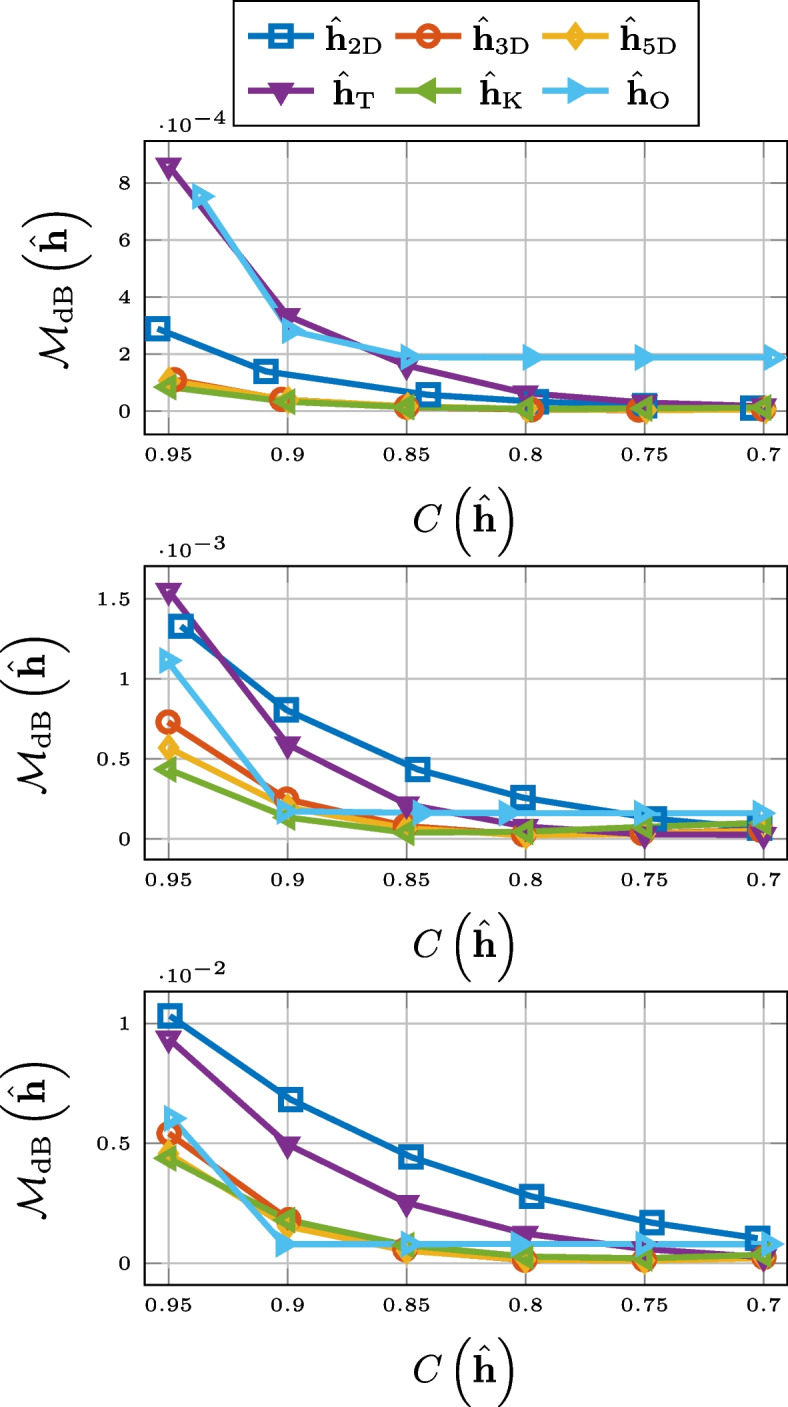
RMSE for the energy decay curve for SMARD (top), AIL (middle), and SAL (bottom) for different compression methods

For the RMSE for the energy decay curve, 2-D matrix approximation and truncation consistently performs the worst, particularly for higher compression rates. For higher compression rates of longer RIRs, Opus performs better, but in most considered scenarios, higher-order tensor approximation and thresholding performs the best. The results are displayed in Fig. 6.

### Echo density

**Fig. 7:**
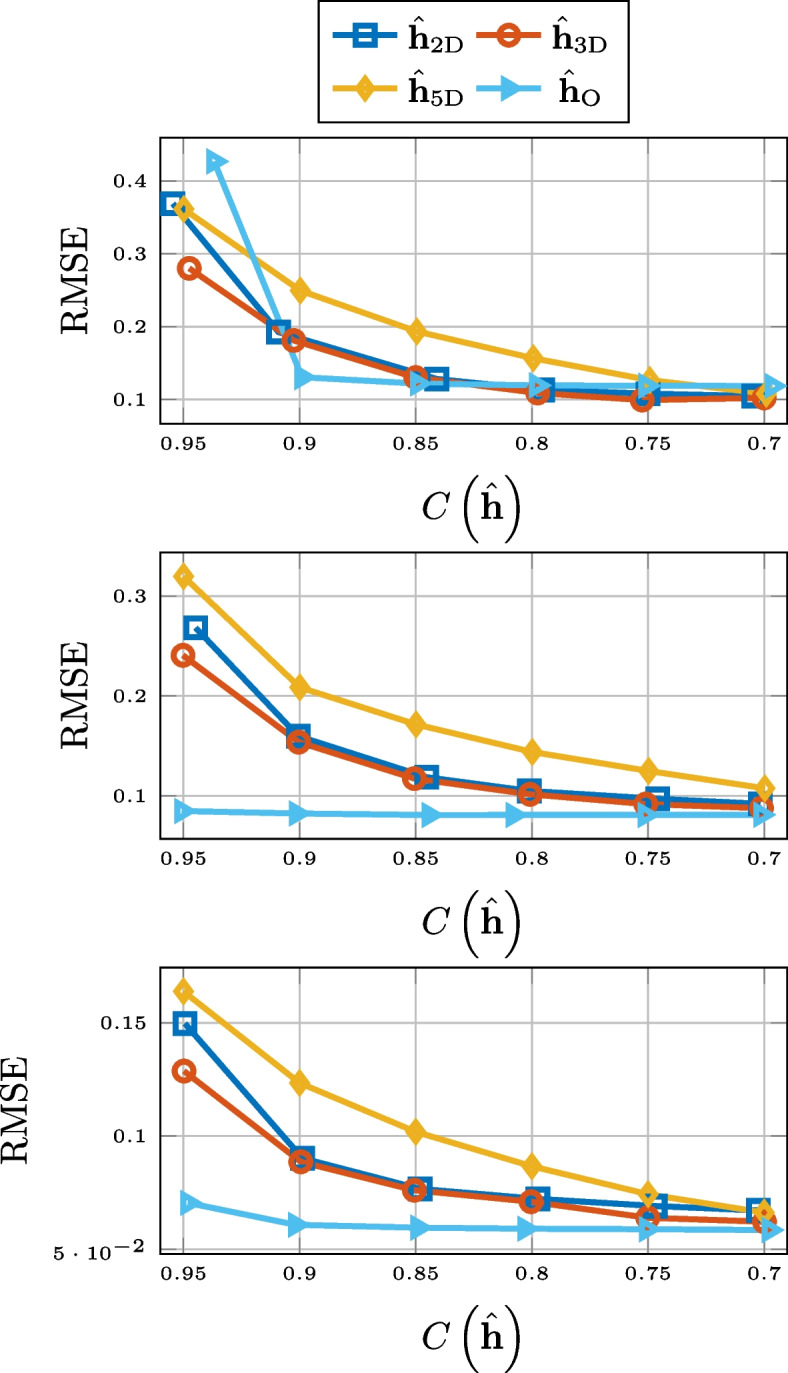
RMSE for echo density for SMARD (top), AIL (middle), and SAL (bottom) for different compression methods

When it comes to preserving echo density, displayed in Fig. 7, Opus is the best of the compared compression methods for longer RIRs. For short RIRs, 2-D matrix approximation and 3-D tensor approximation outperforms Opus, but 5-D tensor approximation does not. Truncation and thresholding are not included in Fig. 7 due to poor performance.

### Early decay time

**Fig. 8:**
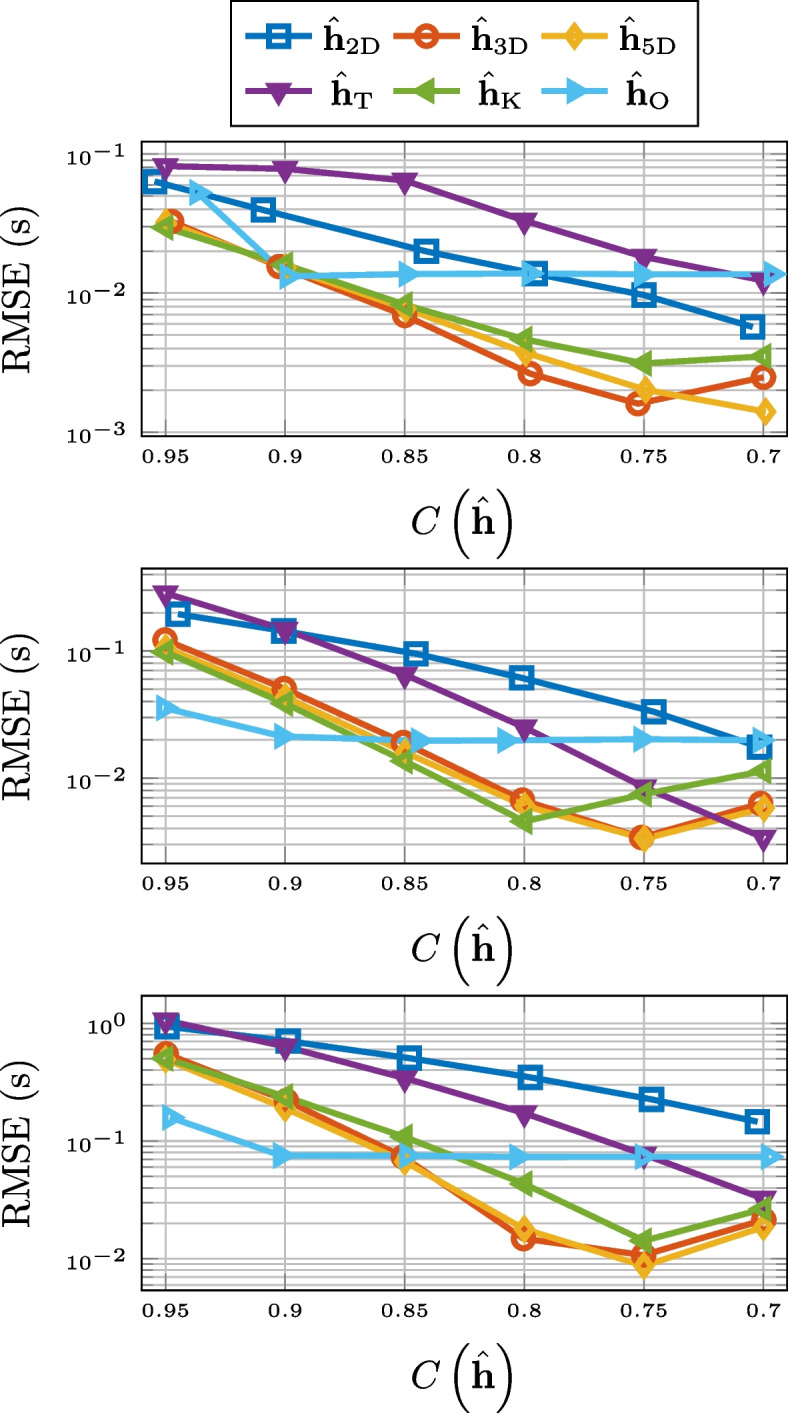
RMSE for EDT for SMARD (top), AIL (middle), and SAL (bottom) for different compression methods

The performance of the different compression methods with respect to preserving EDT is shown in Fig. 8. For this measure, truncation and 2-D matricization performs worst for all considered cases. Opus works better for longer RIRs and for higher compression rates, but for shorter RIRs, and all but the highest compression rates, thresholding, and 3-D and 5-D tensorization are better options.

### TOA of direct component

**Fig. 9:**
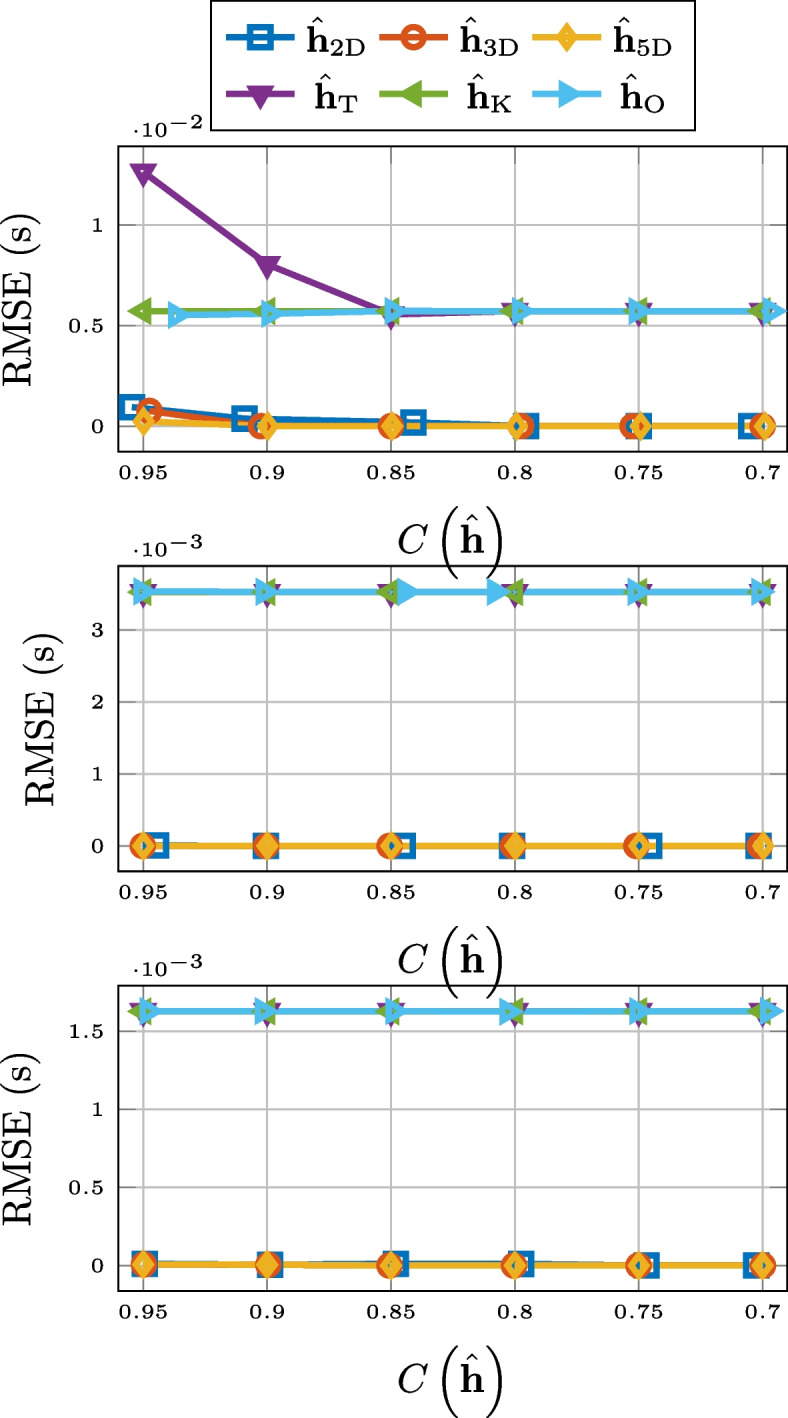
RMSE for TOA of direct component for SMARD (top), AIL (middle), and SAL (bottom) for different compression methods

For the preservation of the TOA of the direct component, there is a clear discrepancy between the compression methods based on low-rank approximation and the other methods. This is evident from Fig. 9, where the results are displayed.

### Center time

**Fig. 10:**
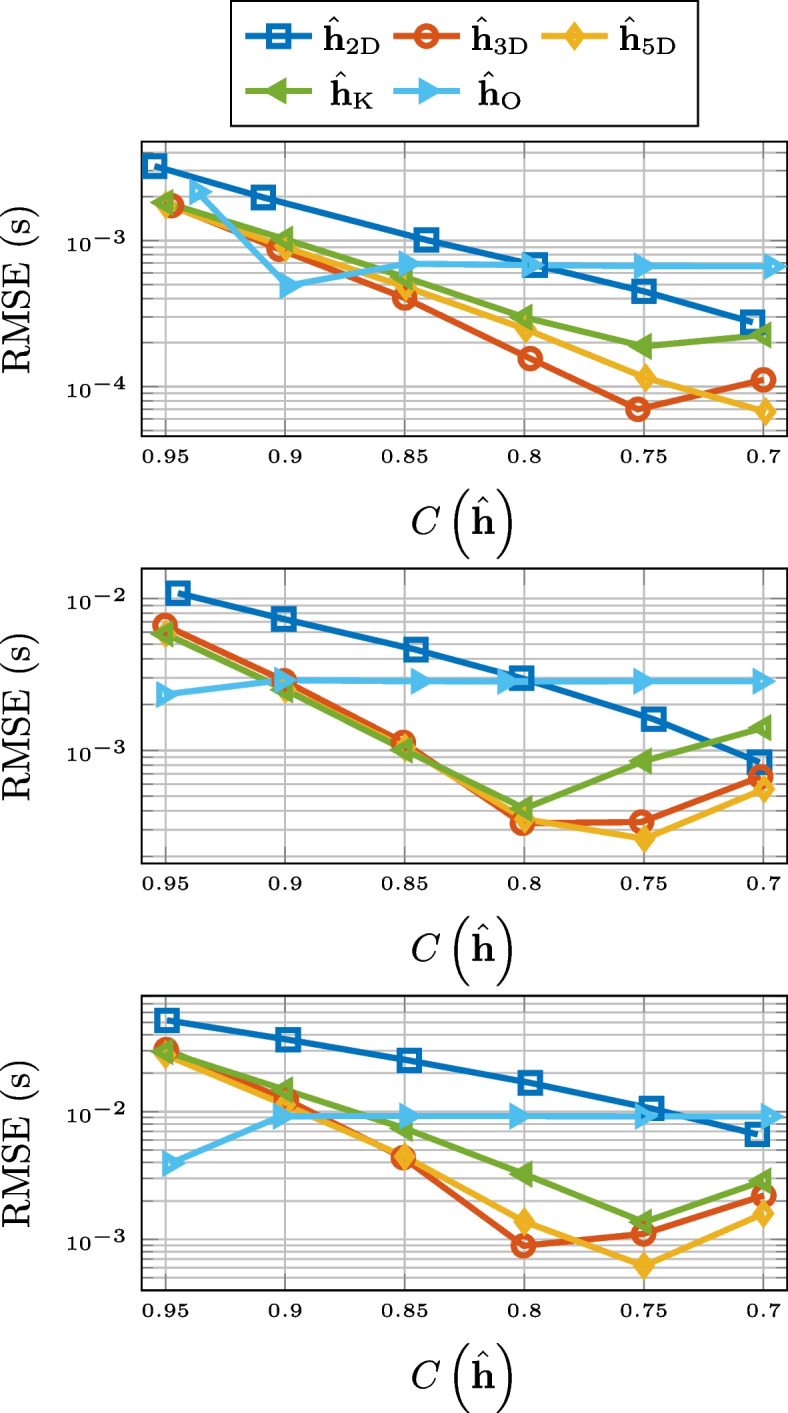
RMSE for center time for SMARD (top), AIL (middle), and SAL (bottom) for different compression methods

In Fig. 10, we see the RMSE for the center time. There it can be observed that the 2-D matrix approximation does not perform on the level of Opus, but thresholding, and the higher-order tensor approximations do, for all but the highest compression rates. The performance of compression by truncation has been left out of the figure.

### Signal distortion

**Fig. 11:**
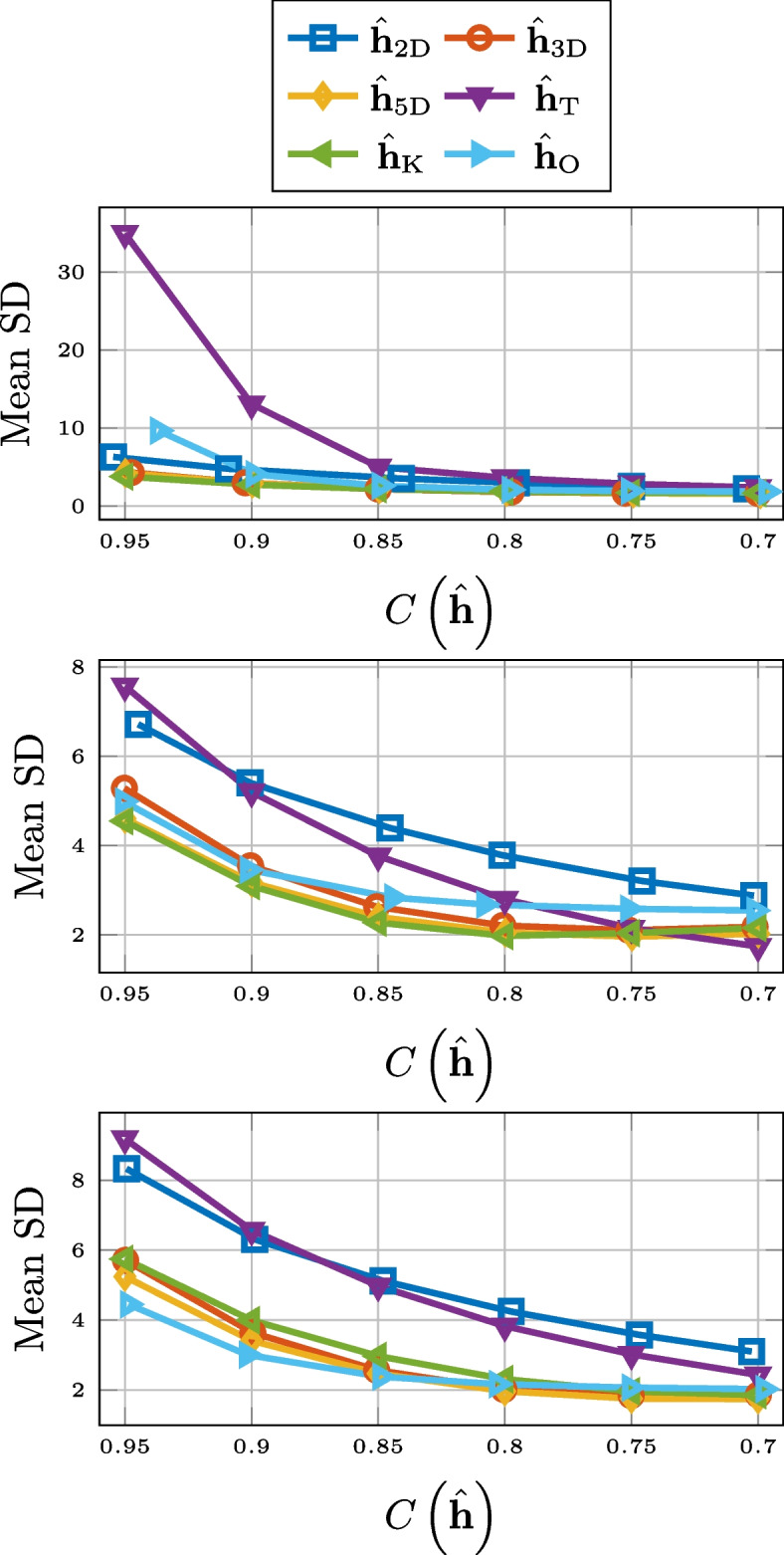
Mean signal distortion for SMARD (top), AIL (middle), and SAL (bottom) for different compression methods

The results for the mean SD are better for the higher-order low-rank methods and thresholding, compared to Opus, except for the highest compression rates for the longest RIRs. This can be seen in Fig. 11. Truncation and 2-D matricization was yet again worse, with the exception for at low compression rates for the RIRs of AIL. Mean signal distortion is the objective measure that best corresponds to the results of the informal listening tests presented in Sect. 5.10.

### ViSQOLAudio

**Fig. 12:**
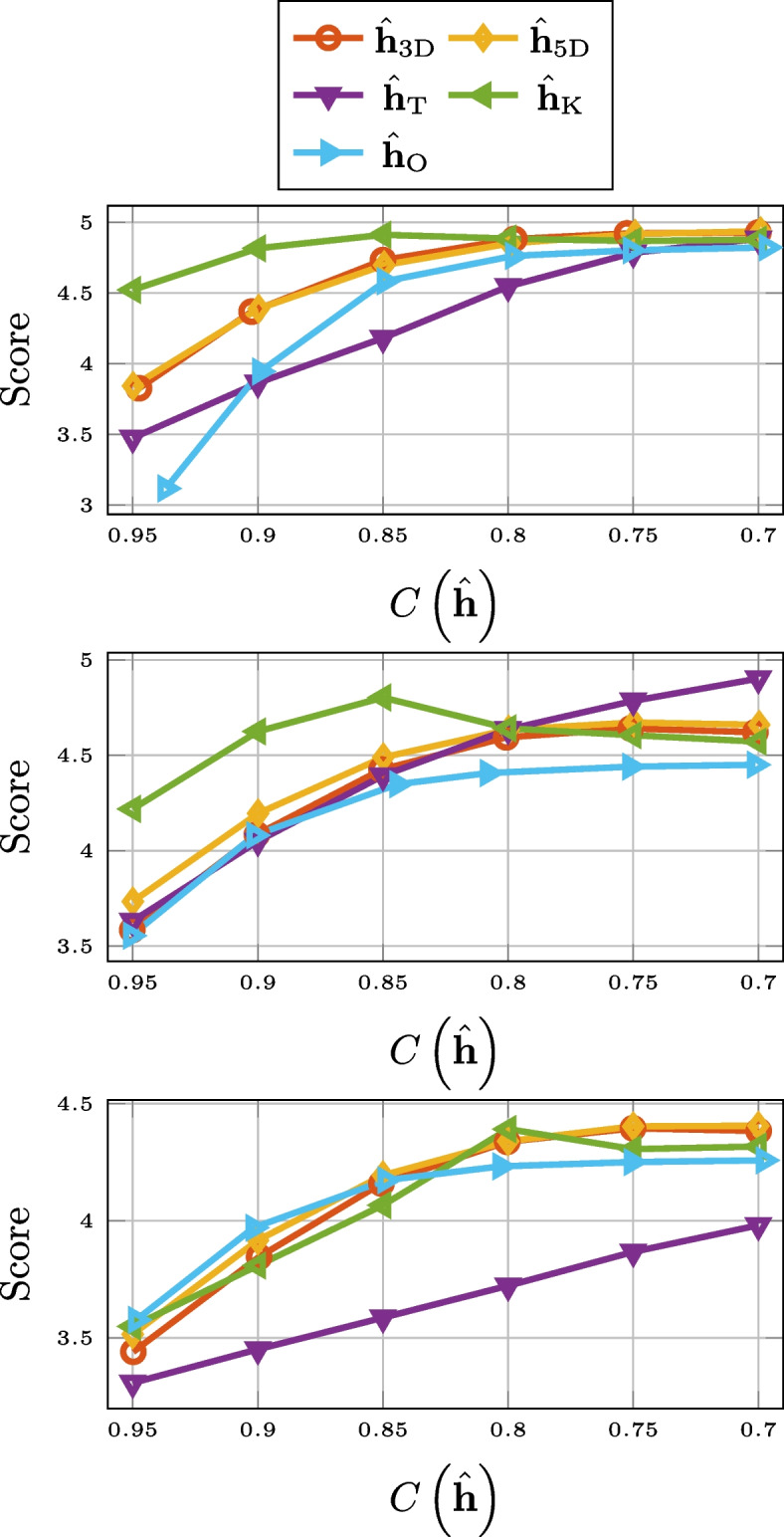
ViSQOLAudio for SMARD (top), AIL (middle), and SAL (bottom) for different compression methods

In Fig. 12, the ViSQOLAudio scores for varying compression rate are displayed. It is only for high compression rates of very long RIRs where Opus is a better option than 3-D tensorization, 5-D tensorization, and thresholding. For ViSQOLAudio, the results for 2-D matricization was left out of Fig. 12 due to poor performance.

### Informal listening tests

An informal listening test was conducted amongst the authors of this paper, with the aim of complementing the results for the objective quality measures reported above. The listening examples used in this informal test are publicly available,^2^ and were created by applying each of the compression methods to one RIR from the SMARD database and one RIR from the SAL database, and subsequently convolving these with two distinct anechoic music signals taken from the set of music snippets used above. A reference output signal was created for each combination of one RIR and one anechoic music signal, by convolving the anechoic music signal with the original RIR without compression. From the results in Figs. 11 and 12, we expect that compression artifacts and quality differences may only become audible for high compression rates, and for this reason the listening examples have been created for the highest compression rate considered before, i.e., $C(\hat{\textbf{h}}) = 0.95$. The listening test results can be summarized as follows.

For the short RIR from the SMARD database, the Opus compression yields a strongly audible loss of brightness in the output signal, which can be attributed to the low-pass filtering operation as part of the compression. Indeed, for very high compression rates only the LPC-based layer of Opus is used, which involves a downsampling to 8 or 16 kHz [48]. This low-pass filtering effect has been confirmed by inspecting the frequency magnitude responses before and after Opus compression, and may largely explain the poor performance of Opus observed in Figs. 11 and 12 for SMARD and $C(\hat{\textbf{h}}) = 0.95$. The compression by truncation results in a slight loss of reverberance, whereas the compression based on thresholding and low-rank approximation do not yield perceptual differences compared to the reference signals.

For the long RIR from the SAL database, the most explicit perceptual artifact consists in a significant loss of reverberance due to the shortening of the RIR tail when performing compression by truncation and by thresholding. A more subtle artifact is a slight loss of dynamic range observed for compression by thresholding. Finally, both the Opus and low-rank-based compression methods exhibit a slight loss of reverberance, which is somewhat more pronounced for the low-rank-based methods and which increases as the dimension *D* of the RIR tensorization is reduced.

## Conclusions

In this work, we have considered different RIR approximation methods for the purpose of RIR compression, aiming to save data storage and accelerate time-domain convolution. It was found that RIR truncation performs worst in almost all scenarios considered and can therefore not be recommended. With the exception of echo density, the RIR compression by thresholding generally preserves well the RIR qualities considered here, compared to the state-of-the-art Opus. For the low-rank approximation methods, 2-D matricization falls short on certain measures, such as mean signal distortion and ViSQOLAudio. The 3-D and 5-D tensor approximations generally outperforms thresholding and they are more robust, as there was no considered scenario or measure where they preformed significantly worse than the other methods, and they perform better than thresholding with respect to the signal-based measures. Much like thresholding, 3-D and 5-D tensor approximations cannot compete with Opus when it comes to preserving echo density, and for the highest level of compression rate, Opus is also better when it comes to preserving EDT and center time. For all other considered measures and scenarios, 3-D and 5-D tensor approximations are as good, or better, than Opus. Add to this the fact that the low-rank tensor approximations are amenable to fast time-domain convolution, and they stand out as the superior choice compared to Opus.

Future research should mainly focus on four open questions. Firstly, investigating whether the promising results for the objective measures considered here will translate into superior performance also in subjective listening tests. Secondly, the fact that the low-rank approximations preserve the TOA of the direct component almost flawlessly indicates that these approximations could be very useful also in the context of spatial RIRs, which needs to be further explored. Thirdly, the occasional discrepancy in performance between the 3-D and 5-D tensorization methods is not yet well enough understood and needs to be further investigated. Finally, a systematic review of the case where $n_{s_1} = n_{s_2} = \dots n_{s_D}$ does not hold true is needed.

## Supplementary information

Supplementary Material 1

## Data Availability

Not applicable.

## Footnotes

1 In previous work [37], we have referred to this as *KMax*.

2 https://github.com/m-jalmby/Informal_Listening_Test_Examples
